# The Flow Dependent Adhesion of von Willebrand Factor (VWF)-A1 Functionalized Nanoparticles in an in Vitro Coronary Stenosis Model

**DOI:** 10.3390/molecules24152679

**Published:** 2019-07-24

**Authors:** Yathreb Asaad, Mark Epshtein, Andrew Yee, Netanel Korin

**Affiliations:** 1 Faculty of Biomedical Engineering, Technion-Israel Institute of Technology, Haifa 32000, Israel; 2 Division of Hematology and Oncology, Department of Pediatrics, Baylor College of Medicine, Houston, TX 77030, USA; 3 Life Sciences Institute, University of Michigan, Ann Arbor, MI 48109, USA

**Keywords:** atherothrombosis, von Willebrand factor, nanoparticle adhesion, drug delivery

## Abstract

In arterial thrombosis, von Willebrand factor (VWF) bridges platelets to sites of vascular injury. The adhesive properties of VWF are controlled by its different domains, which may be engineered into ligands for targeting nanoparticles to vascular injuries. Here, we functionalized 200 nm polystyrene nanoparticles with the VWF-A1 domain and studied their spatial adhesion to collagen or collagen-VWF coated, real-sized coronary stenosis models under physiological flow. When VWF-A1 nano-particles (A1-NPs) were perfused through a 75% stenosis model coated with collagen-VWF, the particles preferentially adhered at the post stenotic region relative to the pre-stenosis region while much less adhesion was detected at the stenosis neck (~ 65-fold less). When infused through collagen-coated models or when the A1 coating density of nanoparticles was reduced by 100-fold, the enhanced adhesion at the post-stenotic site was abolished. In a 60% stenosis model, the adhesion of A1-NPs to collagen-VWF-coated models depended on the location examined within the stenosis. Altogether, our results indicate that VWF-A1 NPs exhibit a flow-structure dependent adhesion to VWF and illustrate the important role of studying cardiovascular nano-medicines in settings that closely model the size, geometry, and hemodynamics of pathological environments.

## 1. Introduction 

Cardiovascular diseases are a major cause of morbidities and mortalities globally [1,2]. Atherosclerosis is a chronic inflammatory blood vessel disease, characterized by the deposition of atheromatous plaques in the inner layers of arteries [2,3]. As a result of plaque buildup on the vessel wall, atherosclerotic sites are characterized by lumen narrowing. Correspondingly, the hemodynamic conditions are considerably different from normal conditions [4]. These sites are characterized by pathological shear stress that can exceed normal levels, reaching over 1000 dyne/cm^2^ [5]. Plaque fissuring or rupture at these sites exposes inner layers of the vessel wall, including collagen type I. Collagen type I is a major constituent of the extracellular matrix within the blood vessel wall and atherosclerotic plaques [6]. Exposure of collagen, among other inner layers, facilitates thrombus formation at the lesion site, a condition termed atherothrombosis. Atherothrombosis can clinically result in life-threatening conditions such as heart attack and stroke [7]. 

**Figure 1:**
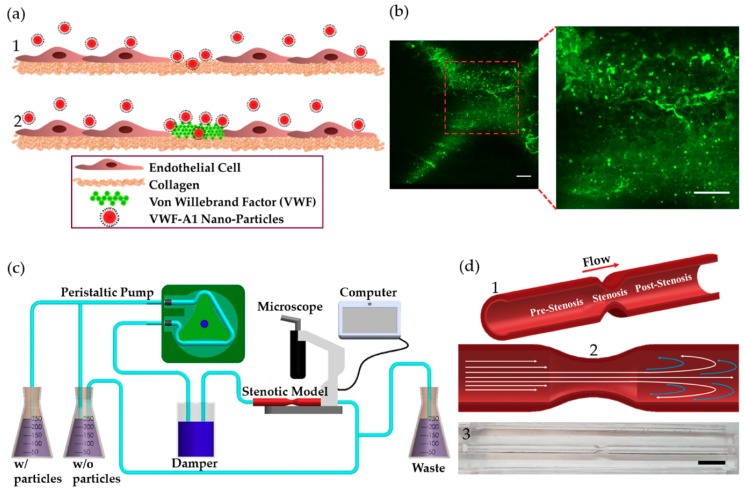
VWF-A1 nanoparticles interactions at injury site and stenotic models. (**a**) A1-functionalized nanoparticles interactions at injury site at different stages of arterial thrombosis: (1) Initially, collagen is exposed (2) Thenceforth, VWF adheres to the exposed collagen. (**b**) Fluorescent microscopy images showing labeled VWF immobilized inside the coronary stenotic model. Scale: 50 µm (**c**) The experimental perfusion system comprising: A peristaltic pump, a damper, containers, and the stenosis models placed under a microscope. 5 µg of A1-NPs were injected into 300 mL dextran solution, having a viscosity of 3.5 mPa·sec, which circulates through a stenosis model via a closed loop flow system. (**d**) The coronary stenosis model. (1) Schematic representation of a coronary stenosis model. (2) Schematic illustration of spatial flow patterns in the stenotic blood vessel. (3) A picture of the PDMS-based 75% stenotic model. Scale: 1 cm.

Arterial thrombosis is a complex cascade that consists of multiple events. These events are orchestrated by numerous factors, including platelets, endothelium, subendothelial matrix, and other hemostatic proteins (e.g., fibrinogen and von Willebrand factor (VWF)) [8]. VWF is a multimeric glycoprotein that is involved in hemostasis and plays a key role in arterial thrombosis. A subunit of mature VWF consists of distinct domains that are linked in the following order (N- to C-terminus): D′-D3-A1-A2-A3-D4-C1-C2-C3-C4-C5-C6-CK [9]. VWF is one of nature’s examples for an integrated mechanical and biochemical responsive molecule. It has a unique structure that enables it to alter its conformation in response to changes in flow conditions. Under pathologically high fluid shear rates, (>10,000 s^–1^), VWF changes its conformation from globular to stretched, uncovering previously shielded binding sites [10,11,12]. VWF anchors to the exposed subendothelial layers (e.g., collagen, Figure 1a) via its A3 domain while its A1 domain interacts with its platelet receptor glycoprotein Ibα (GPIbα) and captures platelets from the blood stream [13,14,15,16]. Studies have also demonstrated that VWF immobilized onto the subendothelial layer undergoes homotypic self-association with soluble VWF multimers, which further facilitates platelet adhesion and accumulation at the arterial injury site [17,18]. Although the exact mechanism of VWF self-association is yet to be fully deciphered, it is clear that multiple domains of VWF participate in this process. 

Localized, controlled drug delivery with nano-carriers to treat cardiovascular diseases is a promising field [19]. The development of functionalized nanoparticles for targeting drugs to sites of vascular injury has received considerable attention [20]. Inspired by the reciprocal interactions along the injury-VWF-platelet axis, different functionalized nanoparticles have been fabricated. For example, nanoparticles decorated with A1, GpIbα, collagen binding peptides, and VWF-binding peptides have been explored [21,22,23]. One promising study in this field is the work of Doshi et al. [20], where platelet-inspired micro-particles that mimic the size, shape, and the flexibility of platelets were decorated with VWF-A1 or GpIbα. The platelet mimetic nanoparticles were tested in a collagen-coated microchannel and showed promising results in targeting growing thrombi under arterial levels of shear stress [20]. Altogether, such nanoparticle formations leverage the physiological features in the arterial thrombosis cascade and may provide optimal drug carrier for targeting athero-thrombotic sites. However, so far, no studies in this field have addressed the targeting of such functionalized particles in models that depict the size and geometry of an arterial stenosis, where hemodynamic conditions may dramatically change. Based on the key role of VWF in the injury cascade, here we examine the spatial adhesion of VWF-A1 coated nanoparticles (A1-NPs) in coronary stenotic models perfused at a physiological flow rate. A1-NPs were fabricated and perfused over stenosis models coated with collagen or a mixture of collagen and VWF, mimicking the initial stages of vessel injury in stenotic vessels. Results in our model show a flow-structure dependent adhesion of the A1-NPs and highlight the importance of studying nanomedicines using biological models that scale to size. 

## 2. Results 

### 2.1. A1-NPs Adhesion in a 75% Arterial Stenotic Model

**Figure 2:**
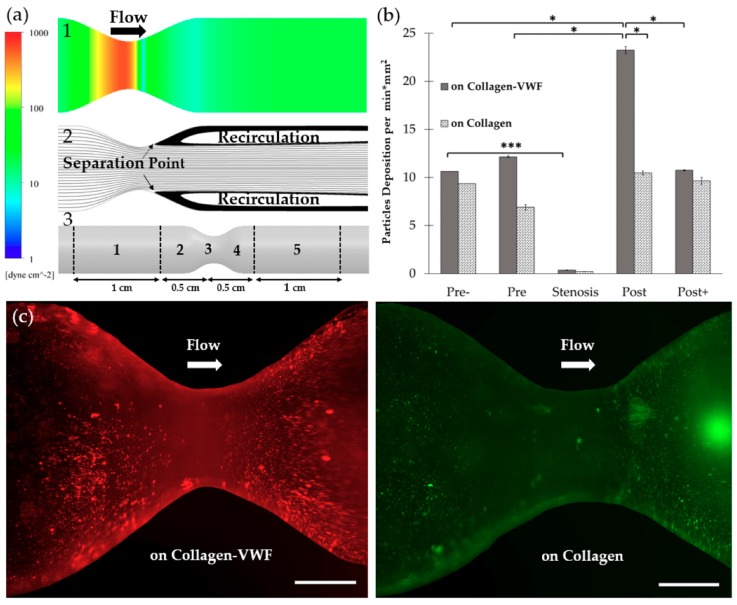
The spatial adhesion of A1-NPs in collagen-VWF vs. collagen-coated 75% stenotic models. (**a**) Computational fluid dynamics (CFD) results and spatial zones along the model. (1) Wall shear stress CFD results for the 75% stenosis model (note: color bar is in log-scale). (2) Streamlines CFD results. (3) A scheme showing the spatial deposition zones along the stenosis model: 1-Pre-, 2- Pre, 3- Stenosis, 4- Post, 5- Post+. (**b**) The normalized A1-NPs deposition at the different zones as a function of model’s coating: Collagen vs. collagen-VWF. (**c**) Representative fluorescent microscopy image of A1-NPs spatial deposition in stenosis model. Left: in a collagen-VWF-coated model (red). Right: in a collagen-coated model (green). Scale: 1 cm.

Models of 75% stenosis were fabricated, coated with collagen or collagen-VWF, and assembled into a closed flow loop for studies of A1-NPs interaction with the vessel wall during the initial stages of arterial injury (Figure 1b,c). The flow rate was set to 200mL/min which corresponds to a Wall Shear Stress (WSS) of 20 dyne/cm^2^ in an unconstricted segment of the model and a Reynolds number of 300. These conditions generate a jet-flow pattern: At the pre-stenosis area the streamlines converge toward the stenosis neck while at the post-stenosis site a recirculation flow is formed, see Figure 1d. Following the perfusion experiments, confocal microscopy images of defined sections in the model were used to quantitatively measure the particle deposition at defined regions. The regions that were examined are the Pre-, Pre, Stenosis, Post, and Post+ as shown in Figure 2a. These different regions are characterized by different levels of WSS and located at different flow structures, see the corresponding Computational Fluid Dynamic (CFD) simulations results shown in Figure 2a–upper panel: WSS, bottom panel: streamlines [21]. 

#### 2.1.1. Effect of the Injury Mimicking Surface: Collagen vs. Collagen-VWF 

**Figure 3:**
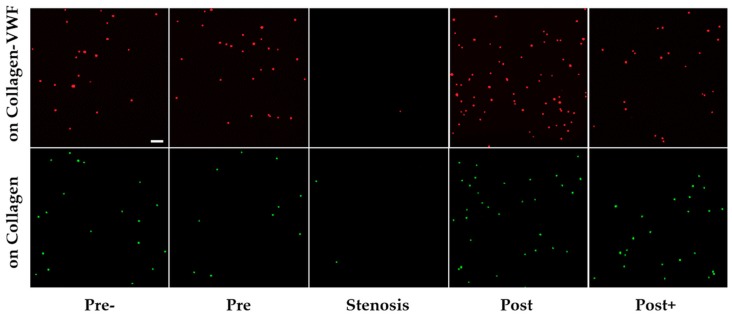
Confocal microscopy images of A1-NPs deposition at different spatial zones in the 75% stenosis model—collagen vs. collagen-VWF coated models. Scale: 1 µm.

To test the effect of the injury mimicking surface, the same A1-NPs were infused over collagen vs. collagen-VWF coated models. Over collagen-VWF coated models, A1-NPs preferentially adhere at the post-stenotic region while only limited adhesion is detected at the stenosis neck (~ 65-fold less particles), see Figure 2b,c and Figure 3 for representative confocal images. The limited adhesion at the stenosis neck correlates with the high WSS inside the stenosis which can reach 600 dyne/cm^2^, see WSS CFD results in Figure 2a. On the other hand, the enhanced adhesion at the post-stenotic region can be attributed to the recirculating flow at this region, see streamlines in Figure 2a. As the recirculation zone is characterized by a low WSS, see Figure 2a, and by long residence times, this may result in increased adhesion. Thus, this adhesion pattern of A1-NPs along the collagen-VWF-coated models is a result of the local flow-structure. However, when infused over collagen, A1-NPs accumulated comparably in the regions flanking the stenosis and failed to adhere to the stenotic neck (Figure 2b). Thus, the A1-NPs do not possess the capacity to adhere to collagen-VWF or collagen coated surfaces at elevated levels of WSS. Although this result is similar for both coating conditions, it is important to note that while the A1-NPs show enhanced adhesion at the post stenotic region in the collagen-VWF-coated models, this does not occur in the collagen coated models. We speculate that one possible explanation for this preferential adhesion may be the higher avidity between A1-NPs and the immobilized VWF, particularly at the post-stenotic recirculation region. 

#### 2.1.2. Effect of A1-NPs Coating Density 

**Figure 4:**
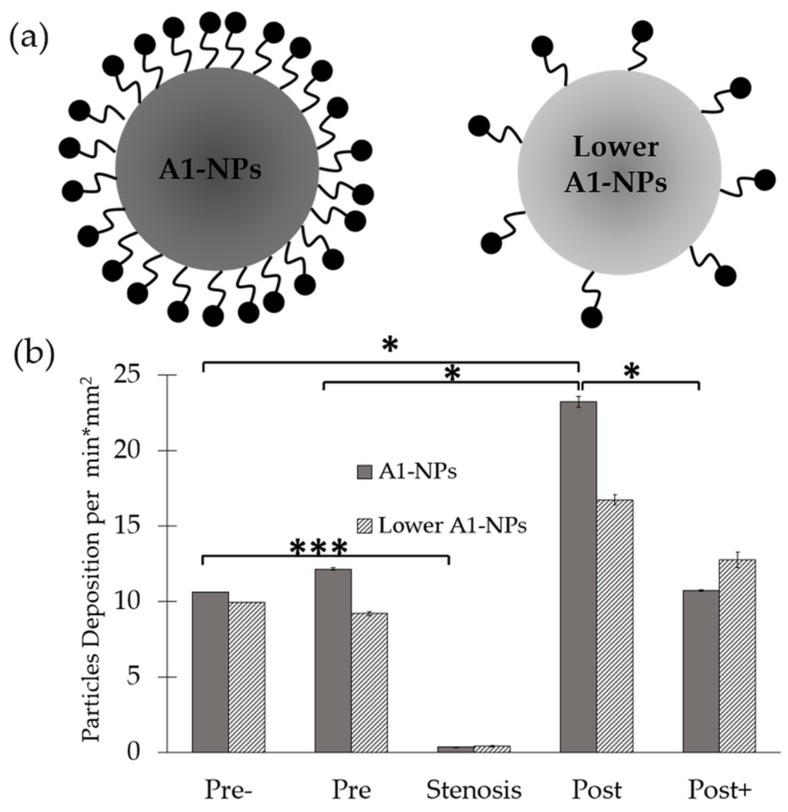
Spatial distribution of A1-functionalized nanoparticles in a 75% stenosis model—effect of A1-NPs coating density on the nanoparticles. (**a**) Schematic representation of A1-NPs and lower avidity A1-NPs. A1-NPs were functionalized with ~5000 copies per µm^2^ while the lower avidity A1-NPs with ~50 copies per µm^2^. (**b**) A graph showing the normalized deposition at different zones along the stenotic model for A1-NPs vs. the lower avidity A1-NPs.

To assess the effect of particle surface-coating density on its adhesion, A1-NPs were fabricated with a 100-fold less A1 on their surface and then infused through the collagen-VWF-coated models. As shown in Figure 4, the lower avidity A1-NPs did not show any preferential spatial adhesion that is statistically significant along the model. Although no statistically significant difference is observed when comparing A1-NPs with lower A1-NPs at a specific spatial zone, a different spatial deposition pattern of A1-NPs and lower A1-NPs can be noted. One possible explanation to these possible differences may be attributed to the different levels of VWF-A1 surface density on the A1-NPs. Since both particles exhibit similar zeta potential values, VWF-A1 surface density may control the adhesivity of A1-NPs.

### 2.2. Effect of the Degree of Stenosis—60% Stenosis Models 

**Figure 5:**
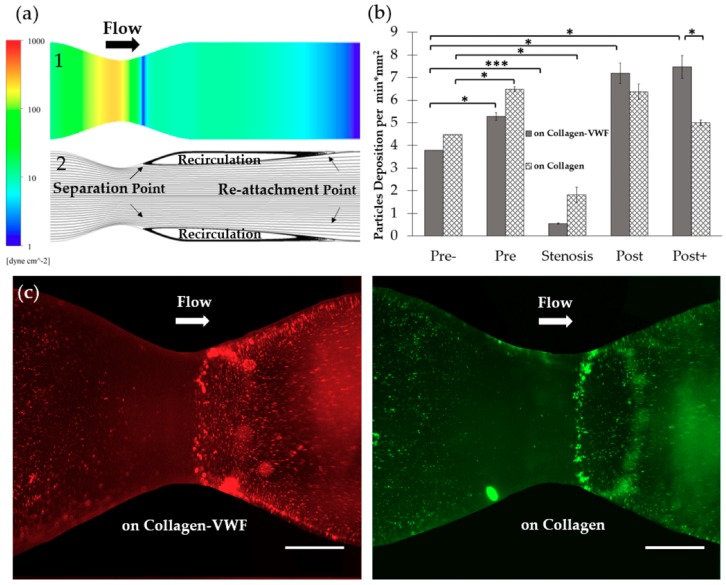
Spatial distribution of A1-NPs in collagen-VWF vs. collagen-coated 60% stenosis model. (**a**) Computational Fluid Dynamics (CFD) of: (1) Shear stress simulation of 60% stenotic model (note: color bar is in log-scale). (2) Streamlines simulation. (**b**) A1-NPs deposition as a function of model’s coating. (**c**) Representative fluorescent microscopy image of A1-NPs spatial deposition in the 60% stenosis model. Left: in collagen-VWF-coated model (red), Right: in a collagen-coated model (green). Scale: 1 cm.

To investigate how the degree of the stenosis affects A1-NPs deposition, a 60% stenosis model was fabricated and coated with either collagen or collagen-VWF. CFD simulations of the flow field in this model revealed a smaller recirculation zone. Although a similar pattern of WSS forms, the WSS levels are lower with the highest WSS reaching 425 dyne/cm^2^, see Figure 5a. As shown in Figure 5b, despite the lower WSS than in the 75% stenosis, almost no A1-NPs adhered to the stenosis neck in both models. Thus, the avidity of the A1-NPs in our tested formulation for collagen or collagen-VWF is insufficient to support adhesion at the stenotic neck where the peak shear stress in the models we tested can reach at least 425 dyne/cm^2^. Additionally, as shown in Figure 5b, there are significant differences between the 60% stenosis models coated with collagen vs. collagen-VWF. In the 60% stenosis model coated with collagen-VWF, A1-NPs deposit at pre, post, and post+-regions (compared to pre-). However, in the collagen coated models the enhanced deposition was noted only at the pre stenotic region compared to pre-. This highlights the important role that VWF plays in the adhesion of A1-NPs at stenotic sites, which produce a flow-structure dependent adhesion. 

Another interesting result to note is the enhanced adhesion of A1-NPs deposition at post+ in collagen-VWF 60% stenosis model, which does not occur at the 75% stenosis model as well as does not occur at the same 60% stenosis models coated with collagen. This may be attributed to smaller recirculation zone in the 60% stenosis than in the 75% stenosis case and the localization of the re-attachment point the post+ region in the 60% stenosis model, as shown in the CFD results presented in Figure 5a. The re-attachment point is an area characterized by low WSS and high WSS gradients, which may alter the VWF conformation and increase its interaction with A1-NPs. To investigate particle deposition under non-pathological and physiological flow conditions, a straight model was fabricated as well. The straight model had a uniform lumen diameter similar to the un-constricted segment of the stenotic model and the shear stress was set to 20 dyne/cm^2^. A1-NPs were perfused through collagen- and collagen-VWF-coated models. By perfusing particles in a straight model, we can investigate particle adhesion without flow disturbance due to stenosis. As shown in Supplementary Figure S1, A1-NPs adhere similarly on collagen- and collagen-VWF-coated models. 

However much less particle adhesion is shown in these uniform un-constricted models compared to pre- zone of the 75% and 60% stenotic models. 

## 3. Discussion

In this study, we investigated the spatial adhesion of VWF-A1 coated NPs inside a stenosis coronary artery model. The study focused on the hemodynamics of the early stages of a rupture to atherothrombotic plaques, which may expose the subendothelial matrix to which circulating VWF adheres. Our results show that localization of A1-functionalized NPs inside the coronary stenotic models is affected by a combination of physical and biochemical factors, highlighting the need to examine NPs deposition while simulating the hemodynamic parameters at thrombotic arterial stenosis.

More specifically, our results show that A1-NPs preferentially adhere to the post stenotic zone of collagen-VWF-coated surfaces in both 75% and 60% stenosis models. This spatial adhesion profile maps with the local flow features, where a recirculation zone exists in the post-stenotic region. Interestingly, when A1-NPs were perfused in collagen-coated models, this flow-structure dependent adhesion was not observed. This difference (Post in Figure 2b and Post+ in Figure 5b) suggests that the deposition pattern depends not only on the flow field but also on the interaction between the VWF-A1 domain and the surface coating.

In our study, we perfuse A1-NPs at a physiological flow rate such that the WSS is 20 dyne/cm^2^ in the straight segment and gradually elevates to 600 dyne/cm^2^ at the stenosis neck [5]. Significantly less particles localized to the stenosis neck. We speculate that the dramatically high shear stress inside the stenotic neck does not allow stable particles’ adhesion with the current formulation of VWF-A1 NPs. To enable deposition at the stenosis neck, particles that are of higher coating density might be used; however, we were not able to form stable A1-NPs with higher levels of surface coating due to self-aggregation. Alternatively, other forms of A1 fragments as well as other coating ligands may be used that would allow nanoparticles to remain stable and that can resist the WSS at the neck of the stenosis. Finally, in this study, spherical nano-polystyrene NPs were used; however, other functionalized particles in terms of polymer matrix, geometry, and size can be utilized [20]. 

In addition to the simplified stenosis geometry we utilized in this study, more complex geometries replicating patient derived coronary arteries may be used in the future. Furthermore, healthy/inflamed endothelial cells can be cultured inside the models, and whole blood maybe perfused. In this study we focused on polystyrene A1-NPs and their adhesion. A1 functionalized drug carriers loaded with potent anti-thrombotic or anti-platelet drugs may be examined in the future as a therapeutic vehicle that can locally inhibit thrombosis. 

## 4. Materials and Methods 

### 4.1. Nanoparticle Conjugation with VWF-A1 and Characterization 

Carboxylate-modified, fluorescently labelled 200 nm particles (ThermoFischer Scientific-Invitrogen^TM^) were conjugated with the VWF-A1 domain via carbodiimide chemistry and avidin-biotin interactions as per Merck-Millipore two-step EDC/Sulfo NHS covalent coupling protocol, with modifications [22]. Particles of 200 nm size were chosen as our nominal particle as they have been widely used in various prior vascular targeting studies [23,24]. Additionally, this size enables single particle detection under fluorescent confocal microscopy imaging, while with smaller NPs single particle detection is more challenging. For their activation, initially, carboxylate-modified nanoparticles were washed with MES buffer (2-(N-morpholino)ethanesulfonic acid, 50 mM, pH 6.0) and subsequently activated with 200 mM EDC (1-Ethyl-3-(3-dimethylaminopropyl)carbodiimide), Thermo Scientific™-Pierce™) and 200 mM sulfo-NHS ((N-hydroxysulfosuccinimide), Thermo Scientific™-Pierce™) for 30 min at room temperature under gentle agitation. Afterwards, the particles were washed to remove unreacted materials by a series of centrifugations. Then, neutravidin (Thermo Scientific™) at a concentration of 2 mg/mL was added to the particles’ suspension. The reaction was allowed to proceed for 2.5 h at room temperature. The particle suspension was then washed and conjugated with biotinylated VWF-A1. For VWF-A1 NPs and lower VWF-A1 NPs preparation, 300 µg/mL and 3 µg/mL coating solutions were used, respectively. The reaction was allowed to proceed overnight at 4 °C. The nanoparticles were washed to remove unreacted materials and reacted with ethanolamine (Fischer Chemical) for 30 min at room temperature to block unreacted carboxyl groups. For the final preparation stage, the particles were reacted with blocking buffer (50 mM Tris, pH 8.0, 0.5% (*w/v*) casein) for 2 h at room temperature. The particles were washed again and preserved in blocking buffer at 4 °C. To assess protein density on particle surface, BCA kit was used obeying manufacturer instructions (Bicinchoninic Acid Kit for Protein Determination, Sigma-Aldrich). Particles surface electrical charge was measured by laser Doppler micro-electrophoresis (Zetasizer Nano-ZS, Malvern Instruments, Malvern, UK). Results have demonstrated that A1-NPs are functionalized with ~5000 copies per µm^2^ while the lower avidity A1-NPs are functionalized with ~50 copies per µm^2^. A1-NPs and lower A1-NPs had a zeta potential of −38.8 ± 0.6 and −38.2 ± 0.3, respectively, as measured in double distilled water (DDW), and diameter (based on number mean) of 293 ± 5 and 308 ± 2, respectively, as measured via Dynamic Light Scattering-DLS (Zetasizer Nano-ZS, Malvern Instruments, Malvern, UK). The zeta potential and size of the particles stored at 4C over one week were examined and shown to remain at the same values as freshly prepared particles.

### 4.2. VWF-A1 Production and Purification 

An expression plasmid was constructed to fuse an N-terminal myc tag, a C-terminal biotinylation site, and a C-terminal FLAG tag to the VWF A1 domain (P1254-L1460). The myc tag was fused to A1 by PCR amplification of VWF cDNA with primers P1, P2, and P3 using Herculase II (Agilent) [25]. The C-terminal fusions were subsequently added by PCR amplification of the myc-A1 fusion with primers P4, P5, P6, P7, and P8. This PCR product was assembled into pMCSG28 (provided by Dr. W. Clay Brown, University of Michigan) using Gibson Assembly Master Mix (NEB); for the assembly, the pMCSG28 vector was PCR amplified with primers P9 and P10. This assembled product fuses a C-terminal His tag and was chemically transformed into Origami B (DE3)pLysS cells (provided by Dr. W. Clay Brown, University of Michigan). The DNA sequence of the A1 expression plasmid (pMCSG28-myc-A1-BioF) was verified by Sanger sequencing and is provided in supplementary text. The primer sequences are provided in Table S1.

*Escherichia coli* hosting the expression plasmid were cultured in lysogeny broth (LB, ThermoFisher) supplemented with 2% glucose (w/v) and 100 µg/mL ampicillin at 37 °C with vigorous aeration until late log phase (absorbance at 600 nm = 0.5 to 0.7). Cells were centrifuged at 4000 g at 4 °C and resuspended in terrific broth (TB, ThermoFisher) supplemented with 0.4 mM isopropyl β-D-1-thiogalactopyranoside (IPTG) and 100 µg/mL ampicillin. Protein expression was induced at 30 °C with vigorous aeration overnight. Cells were subsequently pelleted by centrifugation at 5000× *g* at 4 °C and lysed with CelLytic B (Sigma) supplemented with 100 mM NaCl, 5 mM MgCl_2_, 1X Halt protease inhibitor (ThermoFisher) or 1 mM Pefabloc (Sigma), 0.5 mg/mL lysozyme (Sigma), and 2.5 U/mL Benzonase (Novagen). After 30 min of lysis at 37 °C, the NaCl concentration was brought to 500 mM. Insoluble material was pelleted by centrifugation at 6000 g at 4 °C, and the supernatant was collected for protein purification.

Clarified cell lysate was passed through 2 mL Ni-NTA beads (Qiagen) loaded in a 10 mL centrifuge column (ThermoFisher). The column was washed with 30mL wash buffer (50 mM Tris-HCl, pH = 8.0 + 500 mM NaCl + 0.005% Tween-20 + 10 mM imidazole). A1 was eluted with 10 mL elution buffer (50 mM Tris-HCl, pH = 8.0 + 500 mM NaCl + 0.005% Tween-20 + 300 mM imidazole). The partially purified A1 was reduced with 10 mM dithiothreitol (DTT, ThermoFisher) at room temperature for at least 1 h and subsequently alkylated with 50 mM iodoacetamide (Sigma) at room temperature for at least 1 h in the dark. Unreacted iodoacetamide was inactivated with an additional 20 mM DTT. The reduced/alkylated A1 was dialyzed into TBS-T (50 mM Tris-HCl, pH = 8.0, + 150 mM NaCl + 0.005% Tween-20) with a 20kDa Slide-a-lyzer (ThermoFisher) and concentrated on Amicon Ultra centrifugal filters with a 3 kDa molecular weight cutoff (Millipore). Total protein concentration was measured by spectroscopy at 280 nm. Protein purity was assessed by SDS-PAGE on a 4%–20% tris-glycine gel and staining with ProtoBlue Safe (National Diagnostics). The partially purified A1 was stored at −80 °C.

### 4.3. Fabrication and Coating of Coronary Artery Stenosis Models 

To investigate VWF-A1 particles deposition under realistic hemodynamic conditions, we have fabricated a transparent stenosis artery model. Initially, the model was designed by Computer Aided Design (CAD) software, SolidWorks. Two coronary models of different stenosis ratio were considered, 75% and 60%. The 75% has the dimensions of 4mm diameter in the straight section, 2 mm diameter in the stenotic section and 9.5 cm length. The 60% has the dimensions of 4mm diameter in the straight section, 2.5 mm diameter in the stenotic section and 9.5 cm length. The stenotic ratio was calculated as following:

Stenosis% = (1 − (d_stenosis_/d_straight_)^2^) *100, d = diameter (1)

Clinically, >50% stenotic blockage within arteries is considered moderate since WSS can still not exceed the physiological range, <100 dyne/cm^2^ [5]. In these cases, no surgical intervention is required, and the medical condition is treated with behavioral adjustments. However, 75% stenotic blockage leads to pathological hemodynamics that requires surgical intervention [26]. For this, 60% stenotic model represents a moderate stenotic condition while 75% represents the more severe case. The CAD designs were 3D printed (Form 2 3D printer, Formlabs, USA) to create negative templates for polymer pouring. The models were fabricated from clear resin (compatible for Form 2, Formlabs). To create the 3D stenotic models, unnecessary residues were manually removed, and the model’s surface was polished and smoothed. A mixture of 9:1 ration of ELASTOSIL^®^ RT 601 and RTV-2 silicone rubber (Wacker Chemie AG) was prepared. The mixture was poured into the 3D negative template, allowed to solidify at room temperature, then immersed in acetone to remove the resin template and incubated in a 65 °C oven for at least 48 h to evaporate acetone. The models then were coated with collagen and VWF as thrombogenic surfaces that simulate different stages within arterial thrombosis cascade. Collagen and VWF coatings are well-documented and were extensively used in literature to recapitulate thrombogenic surfaces for investigating platelets adhesion under flow [27]. Several studies have functionalized flow chambers with collagen and VWF to develop platelets adhesion assay under flow and investigate their deposition on thrombogenic surfaces [28]. In this study, we investigated A1-NPs adhesion under flow on collagen and collagen-VWF coated models. Collagen was used to represent the initial stages of arterial thrombosis cascade when subendothelial matrices are exposed, while collagen-VWF coating represents a later initial event that follows collagen exposure. For collagen coating, 100 µL of 3 mg/mL of Human Type I collagen solution (Vitrocol^®^, Advanced Biomatrix) was mixed with 890 µL HBS (HEPES buffered saline, pH 7.2, 20 mM) and 10 µL NaOH (sodium hydroxide, 0.1 N). The collagen was allowed to polymerize inside the models in humidified incubator of 37 °C for overnight. For VWF coating, excess collagen solution was removed and a 250 µg/mL solution of normal plasma VWF (Chrono-log) in DPBS (Dulbecco’s Phosphate Buffered Saline, Sigma-Aldrich) was perfused over the collagen-coated models and then incubated overnight at 4 °C. 

### 4.4. Macro-Fluidics Experimental Setup

To mimic the viscosity of blood, the particles were suspended in dextran solution of 3.5 mPa·sec viscosity (Dextran from *Leuconostoc* spp., M_r_ ~40,000, Sigma-Aldrich). The particles were perfused in a closed-circuit loop via a peristaltic pump (Watson Marlow 530C, Watson Marlow, UK) at 200 mL/min flow rate. The solution then entered a custom-made damper (250 mL bottle with a Thermo Scientific Nalgene 3 port filling/venting cap). The main objective of the pump damper is to adsorb pulsations generated in the operation, thus assuring a stable, constant flow rate. To monitor the adhesion the models were placed under a fluorescent upright microscope (Nikon SMZ25, Nikon Instruments Inc., Melville, NY, USA) and time-lapse imaging was performed. 

### 4.5. Image Acquisition and Analysis 

To quantitatively assess the particles spatial deposition along the model, the later was cut into 5 zones (pre-, pre, stenosis, post and post+) and then imaged using Nikon inverted confocal microscope Eclipse Ti-E (Nikon Instruments Inc., Melville, NY, USA) equipped with 20X objective (N.A. 0.45, Nikon Instruments Inc., Melville, NY, USA) and a EMCCD camera (iXon Ultra, Andor-an Oxford Instruments company, Belfast, Ireland). ANDOR iQ3 Imaging software (Andor-an Oxford Instruments company, Belfast, Ireland) was used for stage movement control and image capture. Images were collected from each zone and analyzed using a custom written MATLAB code. The MATLAB code enables the counting of the number of particles at a defined surface area. To evaluate particle adhesion in each zone, the number of particles in each zone was quantified and normalized by the number of particles at pre- stenosis zone of the same experiment. For the average number of deposited particles, we multiply all normalized values by the average number of deposited particles at pre- zone.

### 4.6. Computational Fluid Dynamics (CFD) 

The simulations were done in ANSYS Fluent^©^ 15.0 applying laminar flow in an axisymmetric model. Two models were used one for 60% and the other for 75% narrowing. Based on convergence studies, both models used about 200,000 tetrahedral elements with uniform inlet. The CFD convergence studies were performed via plotting the wall shear stress along a test line that runs across the model. The inlet Reynolds number based on its diameter was 300 in both cases, and a Newtonian fluid viscosity of 3.5 mPa·sec was used as the fluid. A coupled solver was used, and 1e-12 relative conversion criterion was found to be enough to converge the meshes. The post processing was done in ANSYS CFD-Post suit. 

### 4.7. Statistical Analysis

Data analysis were performed with t-Test: Two Sample Assuming Unequal Variance. The amount of variation within data set was measured by standard deviation (SD), and the hypothesis test was conducted using *P*-value to determine the significance of the results. *P*-value <0.05 is presented using (*), *P*-value < 0.01 (**), *P*-value < 0.001 (***), and *P*-value < 0.0001 (****). All experiments were repeated at least 3 times. 

## Supplementary Materials

The following are available online at https://www.mdpi.com/1420-3049/24/15/2679/s1, Figure S1: Particle Deposition in Collagen vs. VWF-collagen Coated Straight Models, Table S1: Primer Sequences.
